# *Drosophila* SAF-B Links the Nuclear Matrix, Chromosomes, and Transcriptional Activity

**DOI:** 10.1371/journal.pone.0010248

**Published:** 2010-04-20

**Authors:** Catalina Alfonso-Parra, Keith A. Maggert

**Affiliations:** 1 Department of Biochemistry and Biophysics, Texas A&M University, College Station, Texas, United States of America; 2 Department of Biology, Texas A&M University, College Station, Texas, United States of America; Tulane University Health Sciences Center, United States of America

## Abstract

Induction of gene expression is correlated with alterations in nuclear organization, including proximity to other active genes, to the nuclear cortex, and to cytologically distinct domains of the nucleus. Chromosomes are tethered to the insoluble nuclear scaffold/matrix through interaction with Scaffold/Matrix Attachment Region (SAR/MAR) binding proteins. Identification and characterization of proteins involved in establishing or maintaining chromosome-scaffold interactions is necessary to understand how the nucleus is organized and how dynamic changes in attachment are correlated with alterations in gene expression. We identified and characterized one such scaffold attachment factor, a *Drosophila* homolog of mammalian SAF-B. The large nuclei and chromosomes of *Drosophila* have allowed us to show that SAF-B inhabits distinct subnuclear compartments, forms weblike continua in nuclei of salivary glands, and interacts with discrete chromosomal loci in interphase nuclei. These interactions appear mediated either by DNA-protein interactions, or through RNA-protein interactions that can be altered during changes in gene expression programs. Extraction of soluble nuclear proteins and DNA leaves SAF-B intact, showing that this scaffold/matrix-attachment protein is a durable component of the nuclear matrix. Together, we have shown that SAF-B links the nuclear scaffold, chromosomes, and transcriptional activity.

## Introduction

In the eukaryotic nucleus, gene expression is thought to be a multistep process that involves changes in chromatin organization and chromatin structure followed by maturation of the polymerase complex and rearrangements of the transcription unit within the volume of the nucleus. There is an emerging understanding of the connection between nuclear structure and gene regulation[1]. Movement of genes as they are expressed or repressed, stereotyped chromosome domains[2], [3], connections between chromosome linearity and gene expression[4], [5], connections between cohesion and expression[6], [7], [8], *trans*-sensing of homologous chromosomes[9], [10], alterations of chromosome proximity during gene expression[11], and neighborhoods of co-regulated genes [12], [13] all demonstrate an important contribution by nuclear and chromatin organization as gene regulatory networks are activated or inactivated. How these dynamic changes are regulated and the mechanisms by which the are effected are still outstanding questions.

Chromatin is thought to be organized in ordered structures consisting of hundreds or thousands of looped domains fixed at their bases, usually at AT-rich sequence, to a proteinaceous structure known as the nuclear scaffold or matrix[14], [15]. Whatever the effect of changes within the nucleus, scaffold proteins that tether chromosomes to nuclear landmarks are being uncovered as key players – regulators or responders – in gene expression or boundary formation. Recent work enumerates dozens of proteins identified as scaffold/matrix components through biochemical isolation [16]. Several scaffold proteins that bind DNA directly have been identified and characterized, and include topoisomerase, lamin, high-mobility group proteins, and Scaffold Attachment Factors A (SAF-A) and -B (SAF-B). Activities of Scaffold/Matrix Attachment Region (SAR/MAR) binding proteins have been characterized biochemically; although it is still unclear how are they are involved in gene regulation, they have been proposed to contribute to chromatin structure by mediating the attachment of chromatin to the nuclear scaffold thereby folding chromatin into topologically independent loop domains[14], [17]. This view may be an oversimplification of a group of proteins with diverse functions, as many have been shown to affect transcription, replication, RNA processing, and RNA transport[18], [19], [20], [21], [22]. More work is needed to define the role of SARs/MARs and their binding proteins in chromatin remodeling and transcriptional regulation.

One discrete connection between scaffold binding and gene regulation is known from studies of human and mouse SAF-B proteins. SAF-B was independently identified as a protein binding to SAR/MARs, an interaction partner with heterogeneous ribonucleoprotein A1 (therein called *HAP*) [23], and a transcription factor at the *hsp27* and *Estrogen-receptor-alpha* genes (therein called *HET*) [24]. SAF-B misregulation is found in human breast tumor cell lines, suggesting an important role in gene regulation[25], [26], [27], and disruption of one SAF-B paralog results in cell immortalization[28]. Over-expression of SAF-B results in errors in splicing, transcriptional misregulation, nuclear deformation and fragmentation, and apoptosis[18], [29], [30].

Mammals possess two paralogs of the SAF-B family: SAF-B1 and SAF-B2. The two genes are closely-linked and divergently transcribed, arguing for co-regulation by the short (500–700 bp) GC-rich intergenic bidirectional promoter[21]. Both are expressed in most tissues, but have only overlapping cellular localization – SAF-B1 is exclusively nuclear, while SAF-B2 is also found in the cytoplasm. SAF-B1 and SAF-B2 can interact with each other, and localization to different compartments in the nucleus may contribute to complex regulation or separation of function[20]. SAF-B1 has been shown to act as an E-box-binding transcriptional repressor, while SAF-B2 is involved in alternative splicing, and possibly mRNA export, translational control and cytoplasmic signaling[20], [21]. Both have been implicated in chromatin organization, transcriptional regulation, RNA splicing, and the stress response[20], [23], [26]. SAF-B proteins have been shown to interact with RNA polymerase II and a subset of serine- and arginine-rich RNA processing factors (SR proteins) which localize in the nucleus in a speckled pattern[18], [31]. Additionally, SAF-B1 and SAF-B2 interact directly through a C-terminal domain[32]. These observations lend credence to the idea that the matrix is an important scaffold upon which many aspects of genome regulation may occur.

In studies utilizing *in vitro* assays or diploid cell culture, it is difficult to visualize subnuclear structures or to understand details of the dynamic alterations of the matrix in response to alterations of gene activity. Hence, we used the genome sequence of *Drosophila melanogaster* to search for a protein with the characteristics of SAF-B so that we could investigate the matrix in an organism with unique cytogenetic features. We found a sole *Drosophila* homologue to SAF-B, which contains all the conserved domains and motifs of the human homolog. A fusion protein revealed a complex localization within the nucleus, consistent with roles in chromatin structure, transcriptional regulation, and nuclear structure. We have discovered that *Drosophila* SAF-B binds to discrete sites on polytene salivary gland chromosomes, which largely overlap with RNA polymerase II. Alteration of gene expression results in recruitment of SAF-B, and RNAse treatment of nuclei abolishes much, but not all, of the SAF-B chromosomal binding. Deletion of the DNA binding domain eliminates the balance of chromosome association. SAF-B forms weblike continua through salivary gland nuclei, and extraction of soluble nucleoplasmic protein and nucleic acids from diploid and cell culture nuclei leaves a stable matrix of SAF-B. Together, these observations establish *Drosophila* SAF-B as a *bona fide* component of the nuclear matrix that links nuclear structure to gene expression. We discuss a potential role of SAF-B as an integral component of an emerging model of the nuclear matrix as a dynamic, loosely-ordered scaffold upon which genome regulation is organized.

## Results

### CG6995 is homologous to human SAF-B1 and SAF-B2

**Figure 1:**
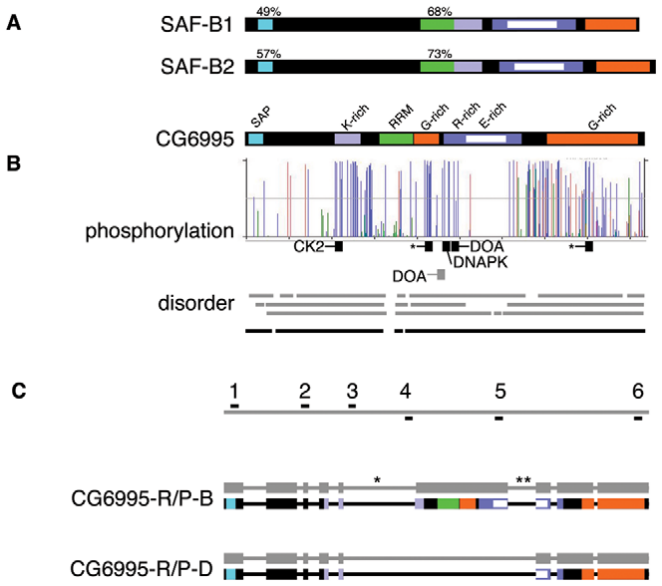
Structure of the gene and gene products of *Drosophila* SAF-B. (A) Human SAF-B1 and SAF-B2 possess the same domains as the *Drosophila* homologue, CG6995. Characterized domains (SAP, RRM) are shown, as well as regions of notable low sequence complexity (K-, G-, R-, and E-rich). (B) NetPhos 2.0 algorithm identification of potential phosphorylation sites, height of bar indicates probability of phosphorylation. Phosphopeptides confirmed in PhosphoPep database are in black, along with putative responsible kinase based on consensus match (asterisks indicate no clear consensus match); gray bar indicates Doa consensus without supporting PhosphoPep support. Three structural determination algorithms (gray bars are PONDR VL-XT, DisEMBL, and IUPred) show extensive predicted intrinsically disordered domains. Averaging scores (black line) shows the only predicted ordered domains are the SAP and RRM domains. (C) Gray bar at top represents genomic DNA, and locations of oligonucleotide primers described in Materials and Methods are shown (1-6). Identified mRNA species (shown as alternating thick exonic and thin intronic gray bars) encode two different protein products (conceptual translation products are shown as thick black bars with colored domains as in (a)). Isoform B is annotated at Flybase, as are two other forms for which we could find no supportive data - isoform A includes intron 5 (asterisk), and isoform C includes introns 5 and 6 (double-asterisk) and is missing exons 1-3 and introns 1-3. Our analyses also identified a novel form, D, which does not possess the RRM, one of the G-rich domains, and contains a shorter R/E-rich domain.

*saf-b* homologues are broadly distributed in eukaryotic organism such as mammals, frogs, birds, arthropods, fungi, plasmodia, and both monocot and dicot plants. Since these proteins are thought to link gene regulation and the nuclear scaffold, we expected SAF-B homologs in all eukaryotes, and were curious whether *Drosophila* contained one or more genes belonging to the *saf-b* family. *saf-b* family members are distinctive because they possess a DNA-binding SAP domain similar to that found in Ku70/Ku80 and Protein Inhibitor of Activated STAT[33], [34], an RNA-recognizing RRM motif, and K-rich, R/E-rich, and G-rich domains. Using these criteria, we used a BLAST search to query the compiled *Drosophila* genomic sequence with the DNA sequence of human SAF-B1 and SAF-B2 homologues. We identified a single gene (CG6995, at cytological band 96A2-3) with identifiable homology throughout the predicted protein, and possessing each of the five recognized domains (Fig. 1A).

CG6995 has regions rich in lysine (18 of residues in a 61 amino acid region are lysine), arginine and glutamate (52 and 47, respectively, in a region of 182 residues), and glycine (40 of 211 residues). These regions contain protein-protein interaction domains[20], [24], [32], [35] but no other ascribed function based on homology to other SAF-B family members.

In addition to the recognized domains and K, R/E, and G regions, CG6995 is rich in serine, threonine, and tyrosine residues, which are capable of accepting phosphorylation modification. Of the 928 amino acid residues predicted from the conceptual translation of SAF-B, there are 122 serine, 33 threonine, and 27 tyrosine residues. Fifty-one residues are predicted to be phosphorylation sites using the *KinasePhos* algorithm[36] and 104 by the NetPhos 2.0 algorithm[37]. Of these, five corresponding phosphorylated peptides have been found *in vivo* using the *PhosphoPep* database (Fig. 1B)[38]. One, with 25 of 42 identified phosphopeptides from the database, corresponds to a Casein Kinase 2 consensus (LLHDEASDDKSIKSVKPANK) with evidence for regulated phosphorylation. Another (SLASQDRPR, 4/42 peptides recovered from database) corresponds to a consensus to the DNA-activated Protein Kinase family (DNAPK). Two sites were identified for the CDC2-Like/LAMMER Kinase Darkener-of-apricot (KESNRARSRRNDDRG and PRHDRERSAKGSQDH), but only a single phosphopeptide of the latter was found in the database. Both potential phosphopeptides are from the region shown to be phosphorylated by CLK2 in humans[18]. Two additional phosphopeptides were identified (SVGGDLKR and RDDSHSLGNKR), but they do not correspond to any described consensus sequence. The protein has a predicted unphosphorylated *pI* of 7.05.

Consistent with the remarkably low sequence complexity, protein structural analyses (PONDR VL-XT, DisEMBL, and IUPred) show extensive regions of predicted disorder. In fact, the only regions with predicted ordered structure are the DNA-binding SAP and RNA-binding RRM domains (Fig. 1B). Others have noted the preponderance of intrinsically unstructured domains in chromatin remodeling proteins and transcription factors, which may play a role in nuclear structure[39].

**Figure 2:**
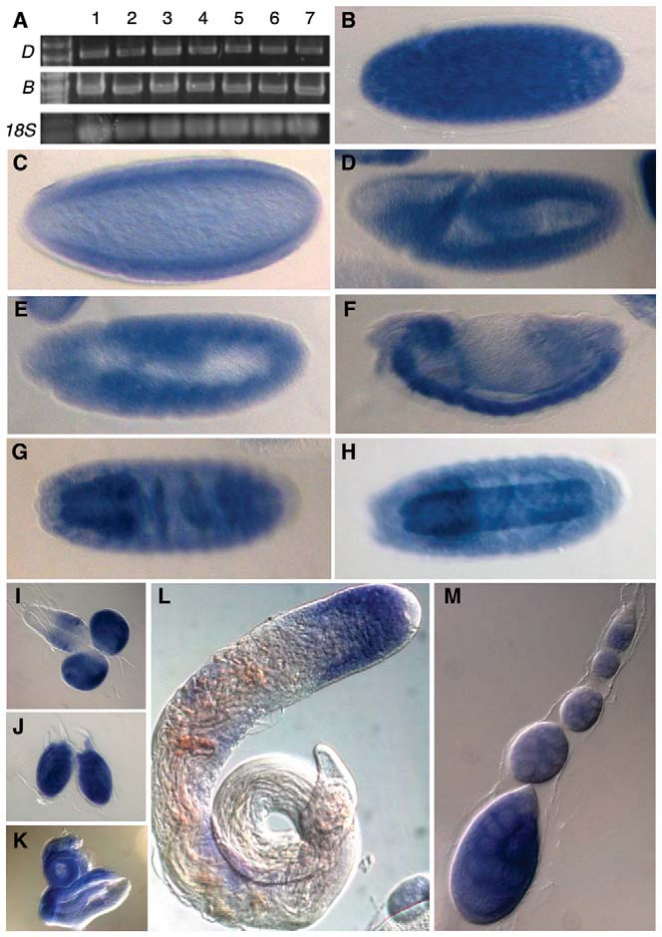
Expression profile of *saf-b*. (A) Reverse-Transcriptase Polymerase Chain Reaction shows expression in all life stages, and in soma (heads) and mixed soma/germ (bodies). Primers 2 and 5 were used for the *B* form, and 2 and 6 were used for the *D* form. *18S* rRNA was used as extraction, reverse transcriptase, PCR, and loading control. Lanes 1–7 are embryos, larvae, pupae, female heads, female decapitated bodies, male heads, and male decapitated bodies. (B–H) embryonic stages showing expression in precellularized syncytial embryos (B), mid-cellularized blastoderm embryos (C), gastrula (D), germ-band elongated (E) and retracted (F) embryos, and late-stage embryos with noticeably intense staining in the central (G) and peripheral (H) nervous system. (I-K) Expression in third-instar larval brains (I), leg imaginal discs (J), and eye-antennal discs (K). (L-M) Expression in the germline of males (L) and females (M). Expression is not detected in the germline stem cells (upper-rightmost tips in both testes and germaria), but is evident in developing spermatocytes, and nurse cells and oocytes.

CG6995 has three reported mRNA splice forms, each with a unique peptide product[40]. CG6995-R/P-B (CG6995 - RNA and Protein - form B) is distinctive because it is the only protein that possesses all of the domains known from mammalian SAF-Bs (Fig. 1C). The existence of the 2784 base pair CG6995-R-B splice form was confirmed with Reverse-Transcriptase-PCR (primer sets 1-6 and 2–5) (Fig. 2A), which codes for a predicted protein with an unmodified mass of 102 kiloDaltons. Using two sets of primers for cDNA construction from adult tissues (separate male and female), mixed-sex larvae, and mixed-sex 0–24 hour embryos, with multiple sets of PCR primers, we were unable to obtain products corresponding to CG6995-R-A (primer sets 2-5 or 3–5) or CG6995-R-C (primer sets for CG6995-R-A or 3–6), and believe them to be aberrant or artifactual splice products erroneously considered mature mRNAs.

We used cDNA from adults to detect the full-length CG6995, and discovered a heretofore uncharacterized splice form of 1744 base pairs (primer set 1–6) (Fig. 1C and 2A). This was confirmed to be a splice form (which we call CG6995-R-D) that retains reading frame and translates to a protein lacking the RRM domain (CG6995-P-D) with a predicted, unmodified mass of 63 kiloDaltons and *pI* of 5.22.

Due to the strong sequence identity between CG6995 to human SAF-B proteins, particularly in the conserved motifs, and in addition to results below showing similarity in expression and localization, we henceforth refer to CG6995 as *saf-b*.

### Expression of SAF-B in *Drosophila*

Human SAF-B1 and SAF-B2 genes are expressed in brain, liver, heart, lung, pancreas, and kidney, suggesting broad or ubiquitous expression[41]. However, expression was limited to a subset of human cancer cell lines[26], [29] and cell types in human testes[20], suggesting potentially regulated expression. We tested whether *Drosophila saf-b* expression is restricted to some cells or whether it is expressed during all developmental stages and in a wide number of tissues. We performed Reverse Transcriptase Polymerase Chain Reaction using primers specific for SAF-B, and detected expression in pooled 0–24 hour embryos, larvae, and adult heads and bodies (Fig. 2A). Primer sets for RT-PCR separately detected both forms, which appear unchanged in relative expression level in the pooled tissue samples.

We considered that expression may be limited or enriched in some tissues, and thus *saf-b* RNA was detected in fixed wild-type embryos and adult tissues by whole mount RNA *in situ* hybridization. We found that *saf-b* RNA is loaded into eggs during oogenesis, and is present in pre-blastoderm embryos. Although tissue-specific expression is not clear in early or mid-stage embryos, by germband extension *saf-b* RNA is present at a higher level in the nervous system than in other tissues (Fig. 2B-H). This enriched nervous system expression persists through hatching and larval molting, and the third instar larval brain and imaginal tissues retain a high level of expression at a time when they are undergoing cell divisions (Fig. 2I–K).

In adult tissues, *saf-b* RNA is found in the testis and ovaries near the apical tip, but distinct from germ cells, thus is low (or excluded) from stem cells, but induced in those cells undergoing premeiotic mitotic divisions (Fig. 2L,M). In testes, expression is clearly absent (or reduced) in gonia, but is evident in the cortically-located spermatocytes. In ovaries, expression begins in stage 3 germaria, at which time premieiotic mitoses have created the 16-cell cyst, and persists through individualized follicles. *saf-b* RNA is loaded into the developing oocyte, consistent with the detection of RNA in unfertilized eggs (data not shown) and embryos prior to the onset of zygotic transcription (Fig. 2B).

Whole mount *in situ* hybridization using RNA probe directed at the RRM-encoding exon only was indistinguishable, indicating that the D isoform was not expressed in a pattern appreciably different from the expression of the full-length B isoform.

### SAF-B is found in two nuclear compartments

We do not possess an antibody specific for *Drosophila* SAF-B, nor do antibodies raised to the human protein cross-react to the *Drosophila* protein (data not shown). Since our goal was to understand where all SAF-B is in the nucleus, regardless of structure or post-translational modification, we constructed a genomic DNA fragment that expressed a fusion protein linking GFP to the C-terminus of SAF-B in order to detect localization of all forms in cells. Such a fusion protein has been used in human cells, and appears to give an accurate reflection of total protein localization[42].

**Figure 3:**
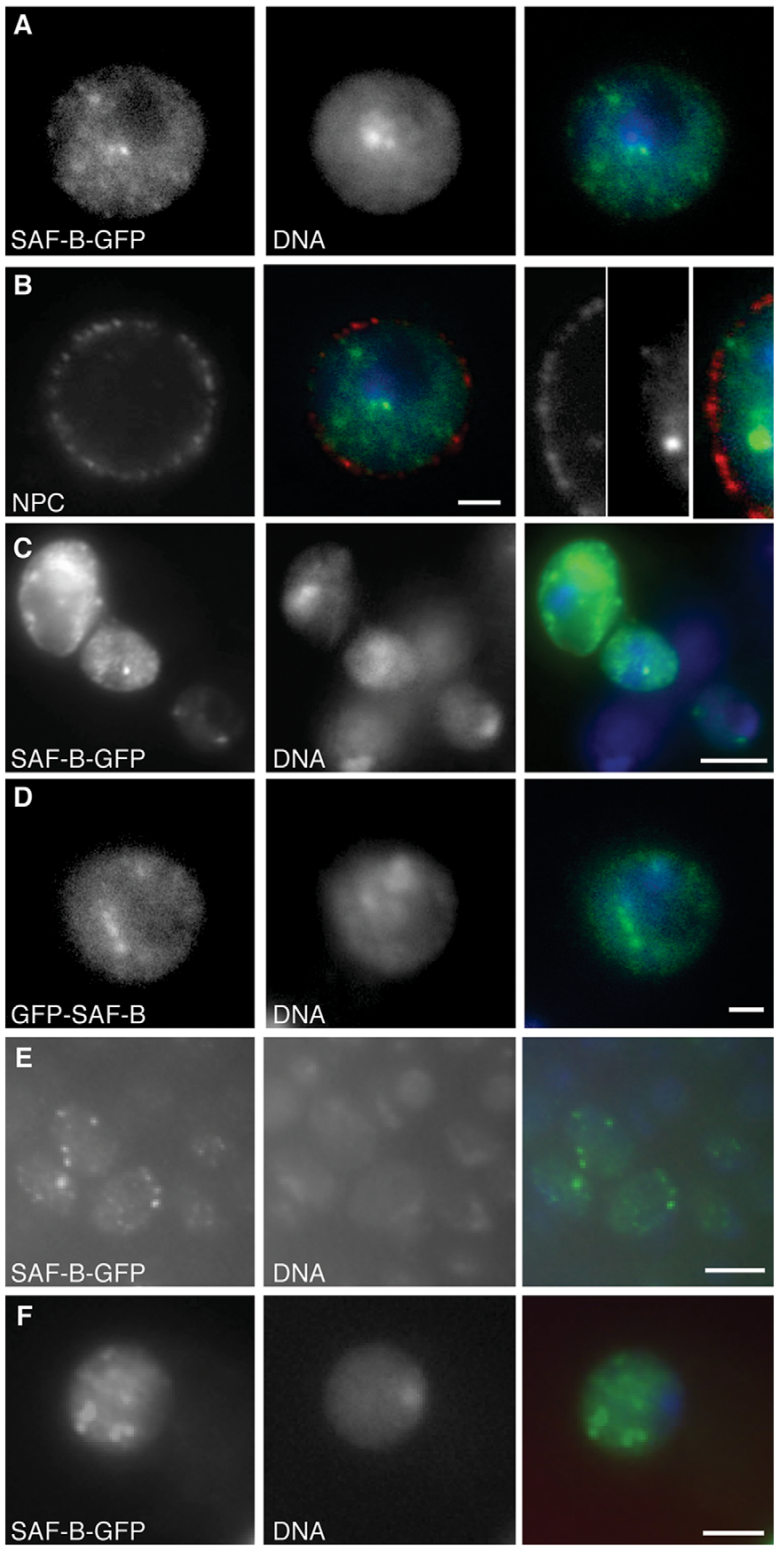
Localization of SAF-B fusion proteins in cells. (A) Carboxy-terminal GFP protein fusion to SAF-B (SAF-B-GFP) in an S2 cell nucleus. Independent channels for immunodetection of SAF-B-GFP and DAPI fluorescence of DNA, and the merge. General nucleoplasmic staining is evident, as well as more intense focal accumulations of protein. SAF-B is not enriched or excluded from the DAPI-bright heterochromatic compartment, but it is clearly excluded from the DAPI-dim nucleolus. (B) Immunodetection of Nuclear Pore Complex proteins p110 and p95, an integral membrane nuclear pore complex, fusion with images from (A), and an image with increased magnification showing no overlap between nuclear pore complexes and SAF-B foci. (C) Immunodetection of SAF-B-GFP and DAPI stained DNA, and the merge. Three S2 cells with different levels of SAF-B-GFP expression all show same nucleoplasmic and focal localization. (D) Amino-terminal GFP protein fusion to SAF-B (GFP-SAF-B) in an S2 cell nucleus. Independent channels for immunodetection of GFP-SAF-B, DAPI fluorescence of DNA, and the merge. Distribution of GFP-SAF-B is identical to that of the carboxy-terminal fusion shown in (A). (E) Distribution of SAF-B-GFP in early pre-determined embryonic nuclei, showing general nucleoplasmic and focal localization, as in S2 cells. (F) Distribution of SAF-B-GFP in larval neuroblast nuclei, showing general nucleoplasmic and focal localization, as in S2 cells and early embryos. Scale bar 2 µm (A, B, D, F) or 5 µm (C, E).

To determine if *Drosophila* SAF-B protein was found in the nucleus, S2 cells were transfected using a plasmid containing this SAF-B-GFP fusion protein under a ubiquitous and constitutive *actin5C* promoter. Cells were transfected and allowed to express for 3 days before they were fixed and analyzed. SAF-B-GFP fusion was found to occupy two compartments within the S2 cell nucleus. First, SAF-B-GFP was found throughout the nucleoplasm, overlapping with chromatin in general, but excluded from the DAPI-dim nucleolus and neither enriched in nor excluded from the DAPI-bright constitutive heterochromatic compartments (Fig. 3A). Second, SAF-B-GFP formed more intense foci in the nucleoplasm (Fig. 3A–D), which did not correspond to any obvious DAPI landmarks. There were many small, less-intense foci, but modally 4-10 larger foci in most nuclei. These foci were also present in living cells (data not shown), and so are not artifacts of fixation. We do not believe that these foci are aberrant nuclear aggregates as a result of over-expression, since we see nucleoplasmic and focal localization even in S2 cells with very low levels of expression (Fig. 3C). Cortical focal localization did not overlap with nuclear pores (Fig. 3B). Identical localization is seen using a GFP-SAF-B (N-terminal) fusion protein (Fig. 3D). Similar partitioning of SAF-B into nucleoplasmic and focal localization has been seen in human cells[20], [22].

S2 cells do not allow an understanding of localization in different cell types. We therefore expressed SAF-B fusions in intact animals under the bipartite control of *actin5C-gal4* and UAS-SAF-B-GFP gene expression system. Localization of SAF-B-GFP appeared as general nucleoplasmic staining with more intense foci, and was similar in cycling embryonic cells and neuroblasts to what we saw in S2 cells (Fig. 3E,F).

Others have reported mammalian SAF-B2 in the cytoplasm[21], [32], and SAF-B C-terminal domains interact with hnRNPs known to shuttle across the nuclear pore. We have also observed weak detection of SAF-B-GFP in the cytoplasm of some of our S2 preparations (data not shown), but cannot rule out artifact since our detection is inconsistent and very close to the background of the immunofluorescence assay.

### Deletion of the DNA binding SAP domain affects subnuclear localization

Mammalian SAF-B can interact physically with the RNA polymerase II C-terminal domain, TAFII15, and the CHD nucleosome remodeling complex[43], and may be recruited to compartments or foci in the nucleus as a result of transcription[18]. However, SAF-B has a SAP domain, a non-sequence-specific DNA-binding domain that shows preference for SAR/MAR DNAs[34], and could be recruited to chromatin compartments as a result of direct binding. It is not known what fraction of chromosome-bound SAF-B is associated with DNA directly, and how much is associated with chromosomes indirectly through nascent pre-mRNA or via protein-protein interactions with the RNA transcriptional machinery.

**Figure 4:**
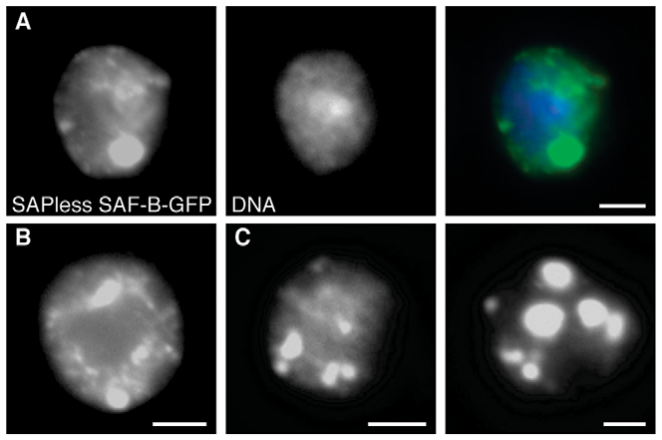
Distribution of SAF-B missing the conserved DNA-binding SAP domain. (A) Truncated SAF-B protein, lacking the DNA-binding SAP domain, fused to GFP, in larval neuroblast nuclei. Independent channels are shown for immunodetection of SAF-B-GFP, DAPI fluorescence of DNA, and the merge. Few smaller foci (as seen in Fig. 3) are often replaced by one or more larger foci. These foci are often connected via continual “threadlike” structures of staining. (B-D) as in (A), SAPless SAF-B-GFP channel only. Scale bar 2 µm.

In order to determine if SAF-B is recruited to DNA as a result of direct DNA binding, we constructed a GFP-fusion to a truncated SAF-B protein that lacked the N-terminal SAP domain. Uniform GFP fluorescence with the same puncta seen for the *wild-type* fusion protein confirmed expression and stability and an active nuclear localization signal, and demonstrated that the SAP domain is not necessary for proper nuclear localization (Fig. 4A), suggesting that a large fraction of localization is not due to DNA contacts through the SAP domain.

We noted consistently enlarged foci of protein in these nuclei (Fig. 4B–D), even when variability in transfection efficiency produced cells with relatively low amounts of chimeric SAF-B protein. The foci seemed in most cases to be associated with decreased general nucleoplasmic localization and by the occasional appearance of continua of GFP fluorescence which connected the bright foci. Not all nuclei showed continua, but we observed that those that did not have continua showed more intense foci, while those with smaller focal staining had more extensive continual filaments (compare Fig. 4B,C to 4D).

### SAF-B forms a network in nuclei and binds to discrete sites on chromosomes

**Figure 5:**
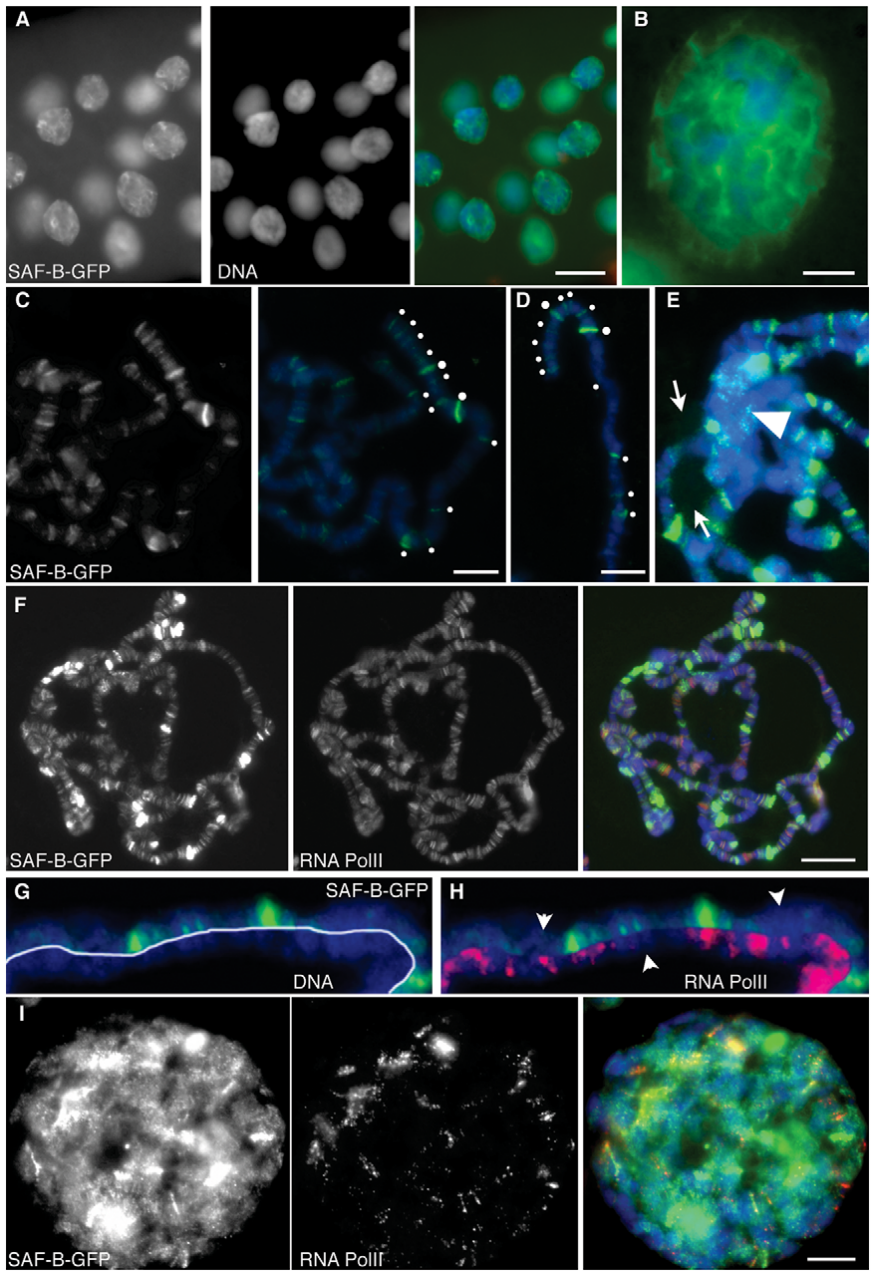
Distribution of SAF-B-GFP in polytene larval salivary gland nuclei. (A) Immunodetection of SAF-B-GFP, DAPI staining of DNA, and the merge. The same nucleoplasmic and focal distribution seen in diploid cells are apparent. Additionally, more intense foci and threadlike continua are seen to connect foci. (B) Increased magnification to show continua. Brighter foci are at confluences of continua. (C) Squashed nuclei showing distribution of SAF-B-GFP, and merge with DAPI-stained DNA. SAF-B associates with specific bands on polytene chromosomes. White dots highlight SAF-B bands at the tip of the *X* chromosome. (D) A different polytene *X* chromosome, labelled as in (C), showing consistency of banding pattern. (E) The chromocenter (arrowhead) of salivary gland chromosomes does not show enhanced or reduced localization of SAF-B-GFP, although some foci are seen, and the nucleolus (arrows) shows only very low level of SAF-B-GFP. (F) Immunodetection of SAF-B-GFP, RNA Polymerase II (Ser2-PO_4_), and merge with DAPI-stained DNA. Extensive areas of overlap of both epitopes are evident, as are bands with detection of only SAF-B-GFP or RNA Polymerase II. (G) Immunodetection of SAF-B-GFP counterstained with DAPI to reveal salivary gland chromosome bands. SAF-B localized primarily to interband regions. (H) Immunodetection of SAF-B-GFP and DAPI staining of DNA as in (G), with immunodetection of RNA Polymerase II (Ser2-PO_4_), showing most bands of SAF-B overlap with RNA Polymerase II, but some bands of only one detectable epitope are apparent (arrowheads). (I) Immunodetection of SAF-B-GFP and RNA Polymerase II (Ser2-PO_4_) in whole-mount salivary gland nuclei, and merge with DAPI-stained DNA. Scale bar 50 µm (A), 10 µm (B–E, I), or 20 µm (F).

We wished to view SAF-B localization in the nucleus at greater detail, and capitalized on the large polytene nuclei of *Drosophila* salivary glands to do so. These cells are interphase, but possess polytenized chromosomes consisting of hundreds of aligned and cohered chromosomes. SAF-B-GFP localization within these nuclei provided additional details of SAF-B structure in nuclei to what we observed in S2 culture and embryonic cells. The immunoreactivity of SAF-B-GFP was found to form a continuous threadlike network (Fig. 5A,B). This is similar to what we saw in some S2 cells for the truncated protein lacking the SAP domain, and may represent the matrix in these cells. Others have documented nucleic-acid-dependent high molecular weight complexes which contain SAF-B[20], although they did not have clear cytological evidence of such complexes. In our studies, there was clear apposition of SAF-B-GFP with DNA, but the majority of SAF-B-GFP intensity did not overlap DAPI-stained DNA, nor was it merely in the nucleoplasm excluded from the chromosomes. Each nucleus had modally three to five foci of increased intensity where these continua converged (Fig. 5A,B,I).

SAF-B in humans binds AT-rich DNAs, but the extent to which SAF-B binds specific genomic loci has not been described, although considerable binding has been reported in promoters of some genes[41]. The DNA binding SAP domain has no known consensus sequence, unlike the AT-hook common in other SAR/MAR binding proteins, and so may bind to sequence even without a high AT constitution. Indeed, binding of human SAF-B to inducible promoters has been described[35], [44]. To better understand the nature of SAF-B-DNA interactions, we squashed salivary glands to free the chromosome arms. Detection of the GFP moiety on these chromosomes revealed that SAF-B-GFP localized to discrete bands on polytene chromosomes, suggesting it binds to specific loci distributed through the genome (Fig. 5C), which are reproducible between nuclei (Fig. 5D). There was not pronounced localization to the nucleolus or to the heterochromatic chromocenter, although the latter had some focal localization (Fig. 5E, arrowhead), consistent with our observations of diploid interphase cells (Fig. 3A,C,D).

Physical interaction of human SAF-B with the transcriptional machinery[24], and genetic interaction with repressed genes [45] suggested that SAF-B might be recruited to active or inactive genes. To address this possibility in *Drosophila*, we performed double localization with SAF-B-GFP and RNAPII(Ser2-PO_4_) antibodies, which detects actively elongating RNA polymerase II. We detected many bands of overlap, but also some bands which contained only one of the two epitopes (Fig. 5F–I). In fact, the intensity of SAF-B detection did not mirror the intensity of RNAPII(Ser2-PO_4_). Less-intense SAF-B bands were found primarily in interbands of the polytene chromosomes, and were found even when RNAPII was not detectable (Fig. 5G,H). In whole-mount nuclei, bands of RNAPII(Ser2-PO_4_) and SAF-B are still evident, and regions of overlap are clear. As with squashed chromosome preparations, it is clear that the most intense foci where continua of SAF-B converge are not simply sites of transcription (Fig. 5I).

**Figure 6:**
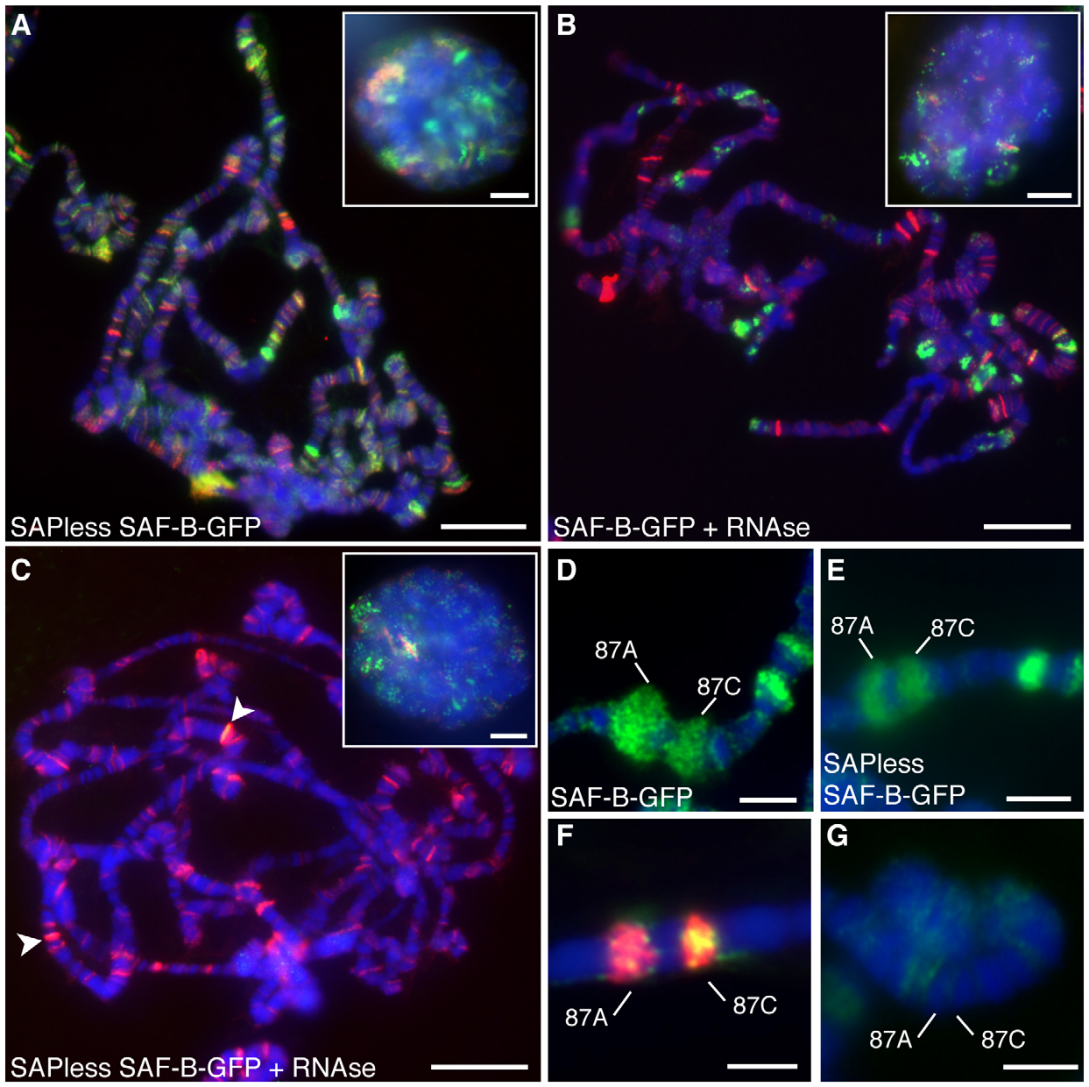
RNA- and transcription-dependent localization of SAF-B. (A) Immunodetection of SAF-B-GFP lacking the DNA-binding SAP domain (green) and RNA Polymerase II (Ser2-PO_4_) (red) merged with DAPI-staining of DNA (blue). (B) Immunodetection of full-length SAF-B-GFP (green) and RNA Polymerase II (Ser2-PO_4_) (red) merged with DAPI-staining of DNA (blue) after treatment with RNAseA. (C) Immunodetection of SAF-B-GFP lacking the DNA-binding SAP domain (green) and RNA Polymerase II (Ser2-PO_4_) (red) merged with DAPI-staining of DNA (blue) after treatment with RNAseA. Arrowheads point to bands of retained SAF-B. Insets in A-C are whole mount nuclei. (D) Immunodetection of SAF-B-GFP at cytological bands 87A-C (green) merged with DAPI-stained DNA (blue), after 15 minute heat shock to induce expression. (E) Immunodetection of SAF-B-GFP lacking the DNA-binding SAP domain merged with DAPI-stained DNA after 15 minute heat shock, showing recruitment of SAPless SAF-B-GFP to the newly transcribed DNA. (F) Immunodetection of SAF-B-GFP (green) at cytological bands 87A-C (green) is reduced after RNAse treatment, although RNA Polymerase II (Ser2-PO_4_) (red) is still present. (G) Immunodetection of SAF-B (green) is negligible prior to heat shock induction of transcription. Scale bar 20 µm or 10 µm (inset images).

The salivary gland nuclear distribution of SAF-B with a deletion that removed the SAP domain looked similar, although subtly different in the number and intensity of SAF-B bands on chromosomes (Fig. 6A), specifically the most intense bands were mitigated. Any retained binding to chromosomes without the DNA binding domain implies that there is another DNA binding domain in SAF-B, or that it is recruited to chromosomes in a DNA-independent fashion. To determine how much of SAF-B is recruited via interactions with RNA Polymerase II or by binding to RNA, we treated nuclei with RNAse prior to immunodetection (Fig. 6B). Most bands disappeared, showing that a large fraction of SAF-B is recruited via direct interaction with RNA, or through some RNA-mediated protein-protein interaction, consistent with the RNA-dependent high-molecular-weight complexes in human cells[20]. A few intense SAF-B bands remained, suggesting some fraction of SAF-B was recruited to DNA via RNA-independent direct DNA binding or protein interaction. To confirm this, we RNAse treated cells expressing SAF-B lacking the SAP domain, and observed that nearly all SAF-B was lost from chromosomes (Fig. 6C). We cannot determine if the few bands that remain on these chromosomes (*e.g.*, arrowhead) are due to protein-protein interactions, particular binding characteristics at these sites, or incomplete RNAse treatment, but our observation that these remaining bands overlap with intense RNA Polymerase II bands suggests the last interpretation.

### SAF-B responds to transcriptional activity

If RNA is responsible for a significant fraction of SAF-B on chromosomes, then induced gene expression should cause new or increased SAF-B binding. Upon heat shock, stereotyped alterations in gene expression reveal new areas of RNA transcription which are visible as decondensed chromosomal puffs [46], [47]. We observed the distribution of SAF-B-GFP in the polytene nuclei of flies before and after a 15-minute heat shock. Prior to heat shock, we did not detect SAF-B at cytological band 87A-C (Fig. 6G). However, SAF-B was directed to puffs of chromatin which were undergoing decondensation and expression (Fig. 6D). Recruitment was not dependent on the DNA binding SAP domain, since a fusion protein lacking the SAP domain behaved the same (Fig. 6E). RNAse treatment reduced the amount of SAF-B-GFP at the heat shock puffs, although RNA Polymerase II remains (Fig. 6F). These data are consistent with our interpretation that much of this protein's association with chromosomes is dependent upon RNA.

### SAF-B is part of the durable nuclear matrix

SAF-B has been considered a major component of the nuclear matrix, determined by its binding to Scaffold/Matrix Attachment Region DNAs. However, biochemical fractionation of human nuclei detected SAF-B in the soluble nucleoplasm and insoluble chromatin fraction, but not in the precipitable matrix fraction[41]. Indeed, others failed to find durable (DNAseI and detergent resistant) nuclear retention common for matrix proteins[22], [48]. However, the purification of SAF-B as a scaffold-associated protein predicted that it would be found in a durable component of the nucleus, either the cortex or an inner network similar to what we saw in salivary gland nuclei. We sought to discover if this was the case in *Drosophila*, as has been shown for other proteins[48], [49].

**Figure 7:**
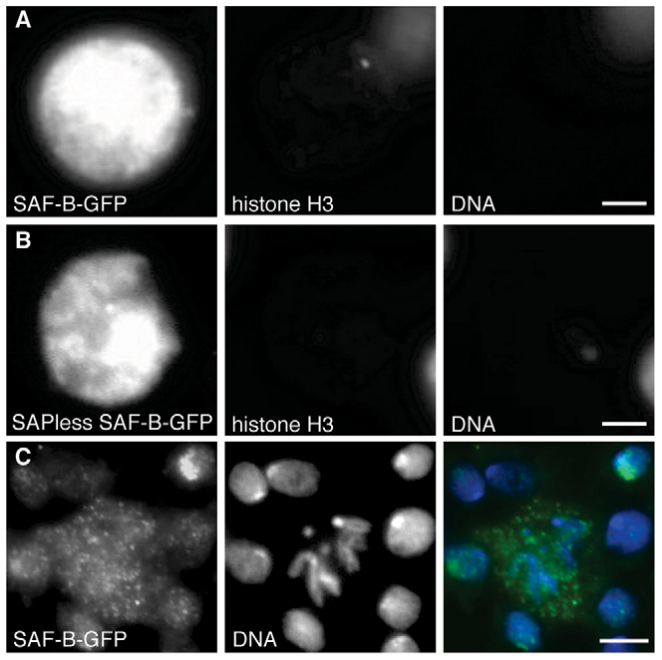
Nuclear extraction reveal SAF-B is a durable component of the nuclear matrix. (A) Immunodetection of SAF-F-GFP and histone H3, and DAPI-staining of DNA after salt, detergent, and DNAseI extraction of mildly fixed nuclei. DNA and histone (a soluble nuclear and chromosome-bound component) are both removed entirely, while the SAF-B is retained. Scale bar 2 µm. (B) SAF-B truncated protein, lacking the SAP DNA-binding domain, is also retained in the nuclear matrix after extraction. Scale bar 2 µm. (C) Immunodetection of SAF-B in diploid mitotic neuroblast cells, DAPI-stained DNA, and merge. SAF-B is found in foci throughout the cytoplasm, and is not detectably associated with chromosomes. Scale bar 2 µm (A, B) or 5 µm (C).

Treatment of S2 cell nuclei with DNAse and detergent removed much of the DNA and all histone H3 (which is not known to be linked to the matrix and thus serves as a positive control for extraction). What remained was a complex of SAF-B-GFP, showing that *Drosophila* SAF-B is included in a DNAse-durable matrix (Fig. 7A). Supportive of the idea that SAF-B is found in the durable matrix, DNAseI-resistant SAF-B-GFP localization did not require DNA binding through the SAP domain (Fig. 7B). We believe that the durable matrix of SAF-B that we observe is maintained through protein-protein or protein-RNA interactions, not through binding of DNA.

Residency in the nuclear matrix predicted that SAF-B should disperse as the nuclear envelope and matrix break down during the cell cycle, rather than be retained with chromosomes packaged as chromatin, a prediction that we confirmed by observing localization in fixed tissues of cycling larval neuroblasts. During mitosis in larval neuroblasts, when the nuclear envelope forms vesicles and no longer contains the chromosomes, we see punctate SAF-B throughout the cytoplasm (Fig. 7C). Discrete sites along chromosome arms or at telomeres are not evident, however low occupancy, dispersed binding, or loss during chromosome condensation might also explain our failure to detect chromosomal foci.

## Discussion

The nuclear matrix (or scaffold) is thought to be a relatively-insoluble scaffold which organizes chromosomes within the nucleus. The matrix has been proposed to organize chromosomes through the cell cycle, and play pivotal roles in organizing replication, transcription, and pre-mRNA processing. However, its existence is controversial to some because it is not observed in living cells, and is only revealed through non-physiological salt treatments or extractions[1], [50]. One putative matrix protein, SAF-B, has been studied since over-expression and biochemical studies have linked roles in gene expression, RNA processing, nuclear structure, and apoptosis [18], [21], [23], [26], [51].

SAF-B was one of the first proteins biochemically identified as a component of the nuclear matrix. It binds to DNA, as was expected, but also to RNA and to other SAF-B molecules[20], [41]. These properties were consistent with expectations for a nuclear matrix protein, and revealed a possible role in coupling nuclear matrix/structure and gene expression. These compelling connections have drawn the attention of many investigators to attempt to understand the role of SAF-B in the nucleus. Despite being a major component of the matrix, SAF-B localization in fixed cells revealed general nuclear localization, and analysis did not allow a detailed understanding of any subnuclear localization, or an understanding of the nature of SAF-B-nucleic acid interactions.

Here, we have provided evidence that *Drosophila melanogaster* has a single SAF-B homologue which shares many features of the two homologues of mouse and human experimental systems. Using fusion proteins and immunofluorescence, we demonstrated features of the putative matrix protein SAF-B in *Drosophila melanogaster*. We observed what appears to be generalized nucleoplasmic distribution with puncta of more intense localization. In these tissue culture nuclei, nuclear extractions showed that SAF-B resides within the matrix. Deletion of the DNA binding domain did not affect nuclear import nor association with chromosomes, but resulted in pronounced large continuous (or aggregated) structures.

The salivary gland nuclei of *Drosophila* are very large, providing us a view of SAF-B in interphase cells. Localization was both cortical and spread through the center of the nucleus, connected in a weblike matrix. This structure may well represent the “biochemically-defined” matrix in these specialized nuclei. Such structures have never been described in mammalian cells where most previous work on SAF-B has been done, but this may be a limitation of resolution in diploid cells of those studies. Indeed, our observations using S2 cell culture did not reveal these structures when using wild-type proteins but they were apparent in the large salivary gland nuclei.

Although we used GFP fusion proteins to detect SAF-B localization, we feel that what we see is an accurate reflection of endogenous SAF-B localization for four reasons. First, the fusion localization in salivary gland cells is similar to that of diploid interphase cells. Second, nuclear GFP is known to be nucleoplasmic, and has not been described to form filaments or territories resembling the SAF-B localization we observe. Third, others have described SAF-B localization in human and mouse diploid cell culture, using direct immunodetection, and HA, FLAG-, GST- and Fluorescent Protein-tagged fusion proteins; all methods resulted in very similar patterns (save for the aforementioned inconsistent cytoplasmic localization)[18], [21], [32], [35], [42]. Fourth, expression of the fusion protein is not lethal to the flies, suggesting that the localization we describe is neither antimorphic nor neomorphic.

Others have used nuclear extracts and fractionation to demonstrate that SAF-B is not part of the nuclear matrix [41]. In contrast, *Drosophila* SAF-B is durable during detergent and nuclease treatment, and forms what appear to be aggregated threads of protein in interphase salivary gland nuclei. We believe that the apparent nucleoplasmic SAF-B of mammals and stable SAF-B of *Drosophila* can be reconciled if we consider that the SAF-B-containing matrix may be a dynamic structure that may assemble or disassemble a subset of total SAF-B, much like the actin, tubulin, or nuclear lamin cytoskeletons. We consider the possibility that the structure of these SAF-B-containing continua or matrices may be ephemeral, subject to post-translational modification, gene activity, or nucleic acid binding[20], and thus only a subset may be stabilized at any time. The scaffold protein SAF-A, which is related to SAF-B in primary structure and forms complexes with SAF-B, is capable of forming strikingly-ordered aggregates when in the presence of DNA or RNA[52]. Whether SAF-B creates, regulates, or is incorporated in these structures has not yet been tested.

A recent report of human SAF-B1 and SAF-B2 chromatin immunoprecipitation with gene promoter regions suggests that SAF-B1 binds to hundreds of genes[51]. This is superficially similar to our demonstration of discrete banded binding to salivary gland chromosomes, however we found that most of the binding in *Drosophila* may be a consequence of transcription, a possibility not considered in that study. Nonetheless, Hammerich-Hille and colleagues did find gross misregulation of many genes, a subset of which overlapped with characterized SAF-B-bound promoters, which provides support for an active role of SAF-Bs in regulating gene expression or chromosome structure.

For a matrix protein to be involved in regulation of gene expression, and be involved in the rearrangements seen in nuclei as new programs of gene expression are induced[53], it would need to be part of the nuclear matrix, be able to pinion chromosomes to the matrix, and reflect alterations in gene expression. We have shown that SAF-B is part of a durable nuclear matrix which is refractory to nuclear extraction, and appears to form interesting and elaborate threadlike continua within some cell types. SAF-B interacts with DNA dependent upon its SAP DNA-binding domain, with other chromosomal loci dependent on RNA, and is recruited de novo upon induction of transcription. How DNA and RNA binding by SAF-B may contribute to the nuclear matrix and coordinate gene expression will continue to be exciting areas of research, particularly with the unique cytogenetic tools available in *Drosophila*.

## Materials and Methods

### DNA constructs

*Drosophila* SAF-B was amplified from wild-type genomic DNA using Polymerase Chain Reaction with primers 1 and 6 (sequences below). The PCR product was cloned into the pENTR/D-TOPO Gateway entry vector according to the manufacturer's instructions (Invitrogen) and the complete sequence was verified by DNA sequencing. The SAP-less form was amplified using primer 6 and CACCATGAGAGCTGAAGGGCTCGACCC. SAF-B sequence was excised from pEntr/D-TOPO and ligated into pAWG, pAGW, pTW, pTGW and pTWG, from the *Drosophila* gateway collection, using the LR Clonase reaction according to the manufacturer's instructions (Invitrogen).

Primer 1: CACCATGCCCGAGGCAGGAAAGAA, Primer 2: GGCTTCCGACGACAAATCTA, Primer 3: TGGATGACGATGGAAACTGA, Primer 4: TGATTGGGTTGCTGATGAAA, Primer 5: GCGCTCCTCACGTATCTTCT, Primer 6: GTAGCGCGACACCGGTC. PCR cycling conditions were: 2-minute 94°C initial denaturation, 35 cycles of 20-second denaturation, 20-second 57°C annealing, and 4-minute 68–72°C extension steps, followed by a final 10-minute final extension.

### *Drosophila *stocks

Flies were maintained on standard cornmeal, yeast, and sugar medium with Tegosept. Crosses were performed at 25°. The wild-type was *yellow* ^1^ *white* ^67c23^. The *gal4* drivers used in this study were: *w* ^1118^; *P*{*w^+mC^ = Sgs3*-*GAL4*.*PD*}TP1 (Bloomington Stock 6870) and *y* ^1^ *w* ^*^; *P*{*w^+mC^ = Act5C*-*GAL4*}25FO1/*CyO*, *y* ^+^ (Bloomington Stock 4414). All fly lines are available from the Bloomington *Drosophila* Stock Center (http://flystocks.bio.indiana.edu).

### Reverse-Transcriptase PCR

Total RNA from embryos, third instar larvae and adult flies of wild-type flies was isolated by lysis and homogenization in TriZOL (Invitrogen), followed by chloroform/isopropanol extraction, ethanol precipitation, and resuspension in DEPC-water. Reverse Transcription was done following the manufacturer's instructions (New England Biolabs). Subsequent PCR amplification of the *18S* rRNA using primers GACTACCATGGTTGCAACGGG and TTCGTCACTACCTCCCCGAG served as control. PCR cycling conditions were: 2-minute 94°C initial denaturation, 35 cycles of 20-second denaturation, 20-second 57°C annealing, and 45-second 68–72°C extension steps, followed by a final 10-minute final extension.

### RNA *in situ* hybridization

Unstaged (0-24 hour after egg deposition) embryos were collected from apple juice agar collection bottles, bleach dechorionated, fixed, and processed for *in situ* RNA detection[54]. Ovaries, brains, imaginal discs, and testis were dissected form larvae or adults in Phosphate Buffered Saline and fixed for 15 min with 4% paraformaldehyde in phosphate-buffered saline. Digoxigenin-labeled antisense RNA probes directed at CG6995 were made by transcribing PCR-amplified DNA using genomic DNA as template and primers TAATACGACTCACTATAGGGATGACCGAGGCAGGAAAGAA and ATTAACCCTCACTAAAGGGAGTAGCGCGACACCGGTC (which include the T7 and T3 RNA polymerase promoters, respectively [55]).

### S2 cells transfection

S2 Schneider cells were grown in Schneider's medium (GIBCO), 10% Heat-inactivated fetal bovine serum (GIBCO) and 50 µg/ml each of penicillin and streptomycin (GIBCO). S2 cells were transiently transfected by calcium phosphate precipitation with pAWG or pAGW containing SAF-B, incubated for 3 days, and analyzed by immunofluorescence or nuclear matrix extraction.

### Nuclear matrix extraction

Cells were washed twice in Phosphate-Buffered Saline (PBS) and extracted in CSK buffer (100 mM NaCl, 300 mM sucrose, 10 mM PIPES pH 6.8, 3 mM MgCl_2_, 1 mM PMSF, 0.5% Triton X-100, and 20 units/mL RNAse inhibitor). After 10 min on ice, the buffer was removed by aspiration. Extractions was carried out by adding Extraction buffer (250 mM ammonium sulfate, 300 mM sucrose, 10 mM Pipes, pH 6.8, 3 mM MgCl_2_, 0.5% Triton X-100, 1 mM PMSF, and 20 units/ml RNAse inhibitor) for 5 min at 4°C. Extraction buffer was replaced with Digestion buffer (50 mM NaCl, 300 mM sucrose, 10 mM Pipes pH 6.8, 3 mM MgCl_2_, 0.5% Triton X-100, 1 mM PMSF, 20 units/mL RNAse inhibitor, and 200–500 units/mL RNase-free DNase) and incubated for 60 min at room temperature. The digestion was terminated by replacing Digestion buffer with Extraction buffer. The slides were then processed for immunofluorescence.

### Immunofluorescence and Microscopy

For S2 cells, cells were fixed with 4% paraformaldehyde at 37°C for 30 min[56], and for nuclear matrix extractions, cells were fixed with 2% paraformaldehyde at room temperature for 15 min. Fixed cells were washed, permeabilized in 0.2% Triton X-100 for 10 min, blocked for 30 minutes with bovine serum albumin, and incubated with primary antibody at 4°C overnight. Primary antibodies were removed and secondary antibodies were incubated overnight. Primary antibodies: anti-GFP (Santa Cruz) at 1:200, anti-Nuclear Pore Complex protein (Covance) at 1:200, anti-RNA Polymerase II (Ser2-PO_4_) (Abcam) at 1:200 and anti-H3K4 (trimethylated) (Upstate). Secondary antibodies: FITC-conjugated anti-rabbit goat IgG and FITC-conjugated anti-mouse goat IgG (Jackson Immunoresearch), TRITC-conjugated anti-mouse goat IgG and TRITC-conjugated anti-mouse goat IgG (Jackson Immunoresearch). All secondaries used at 1:200. DAPI (1 ng/mL) was added to Vectashield (Vector Labs) as mounting medium for visualization of DNA.

For RNAse treatment of the whole mount salivary gland nuclei, glands were dissected in PBS and incubated in TBS (10 mM Tris-HCl, pH 7.15, and 150 mM NaCl) plus 50 µg/ml RNAseA for 45 min at room temperature. The glands were transferred to TBS/0.05% Tween 20 for 5 min and then fixed in formaldehyde fixative solution (PBS with 1% Triton X-100, with 3.7% formaldehyde freshly added). For RNAse treatment of squashed salivary gland chromosomes, glands were dissected in PBS and incubated in PBS with 0.1% Triton X-100 for 2 minutes, then were transferred to PBS with 0.5 mg/ml RNAseA for 8 min.

For polytene chromosome squashes, heat shocks were done for 15 min at 37°C [57]. Squashes were washed in PBST (PBS with 0.1% Tween-20) on ice. Antibodies in PBST supplemented with 0.1%–0.5% BSA were added and allowed to incubate overnight at 4°C. Slides were washed in PBST secondary antibodies in PBST incubated 1.5 hours at room temperature. DAPI (1 ng/mL) was added to Vectashield (Vector Labs) as mounting medium for visualization of DNA.
